# Speech-sound memory consolidation over a daytime nap in adults with and without developmental language disorder

**DOI:** 10.3389/fnbeh.2026.1903903

**Published:** 2026-09-30

**Authors:** Ali Solbi, Rahulkrishna Gurram Thimmugari, Anne L. van Zelst, F. Sayako Earle

**Affiliations:** 1 Department of Communication Sciences and Disorders, University of Delaware, Newark, DE, United States; 2 Department of Rehabilitation Medicine, Georgetown University, Washington, DC, United States; 3 Department of Communication Sciences and Disorders, Florida Atlantic University, Boca Raton, FL, United States

**Keywords:** consolidation, developmental language disorder (DLD), perceptual learning, sleep, speech perception, stabilization

## Abstract

**Introduction:**

Sleep plays a critical role in memory consolidation, yet its contribution to the formation of perceptual-linguistic representations remains only partially understood. In the current study, we examined sleep spindles and slow oscillations (SOs) in relation to offline changes in speech-sound memory in adults across a wide range of language ability.

**Methods:**

Twenty-nine monolingual English-speaking participants completed a perceptual training task contrasting retroflex /ɖ/ and dental /d̪/ consonants, followed by a polysomnography-monitored nap.

**Results:**

Slow spindle density was positively associated with perceptual discrimination performance following a nap, whereas slow oscillation (SO) density was negatively associated. SO-slow spindle coupling strength was significantly correlated with language difficulty amongst those with developmental language disorder, such that those with a lower synchronization index were observed with greater difficulties in language ability.

**Discussion:**

Together, these findings point to the potential relationship between sleep and perceptual-linguistic representation, and developmental language disorder.

## 1 Introduction

Memory consolidation is a dynamic process through which newly acquired information is progressively stabilized and reorganized. These transformations are reflected in sleep physiology, particularly in slow oscillations (SOs) and sleep spindles that occur during non-rapid eye movement (NREM) sleep (Diekelmann and Born, 2010; Rasch and Born, 2013). Prior work has consistently demonstrated a positive association between memory performance, SO, and spindle power/density during NREM sleep, specifically with cross-frequency coupling between the up-state of SOs and fast (12–15 Hz) spindles, and between the down-state of SOs and slow spindles (Clemens et al., 2005; Diekelmann and Born, 2010; Stickgold and Walker, 2005; Mölle et al., 2011). Although behavioral evidence for consolidation in speech-perceptual learning is rapidly expanding, the extent to which these neural markers of sleep-dependent consolidation relate to changes in speech-perceptual learning over time remains under-explored.

Sleep-related consolidation effects vary as a function of both sleep stage and memory system. During NREM sleep, declarative memory consolidation is supported by hippocampal reactivation, which promotes systems-level reorganization and the gradual integration of newly encoded information into distributed cortical networks (Born and Wilhelm, 2012; Gais and Born, 2004). In contrast, procedural memory appears to benefit from both NREM and REM sleep (Ackermann and Rasch, 2014; Fischer et al., 2002), although the mechanisms underlying its sleep-dependent consolidation are not as well understood (Prehn-Kristensen et al., 2009; Tucker et al., 2006). Speech-perceptual learning is thought to engage both declarative and procedural memory systems (Chandrasekaran et al., 2014; Earle and Qi, 2022), leaving open questions about how sleep contributes to changes in speech perception.

A substantial body of research has examined sleep’s role in language learning across the lifespan, particularly for vocabulary and grammar (Gomez et al., 2011; James et al., 2017). However, findings related to speech-sound learning have been more mixed. Some studies show that talker-specific perceptual learning remains stable over 24 h (Eisner and McQueen, 2006), while others report improvements following daytime rest independent of sleep (Roth et al., 2005). In synthetic speech learning paradigms, sleep has been shown to restore performance declines observed during wake and protect newly learned mappings from forgetting (Fenn et al., 2003). Similarly, for nonnative speech contrasts such as the Hindi dental–retroflex distinction, overnight sleep—but not equivalent wake intervals—has been associated with stabilization against interference and improved identification of novel talkers (Earle and Myers, 2015a, 2015b). Notably, improvements in identification do not always correspond to gains in discrimination. Identification requires a listener to assign a single token to a learned category and therefore draws on the stored representation of that category, whereas discrimination requires only a same-different judgement between two tokens and can in principle be performed on acoustic detail alone. Thus, these tasks index qualitatively different perceptual skills, and may point to potentially distinct effects of sleep on task performance (Earle and Myers, 2015a, 2015b). More recently, cross-talker generalization of learned lexical tones has been linked to SOs and fast spindles, whereas sleep did not significantly alter perception of the target contrast in trained talkers (Qin et al., 2025). Taken together, this literature converges on the view that a post-training interval containing sleep benefits speech-sound learning, but it does not specify which physiological events result in perceptual gains, nor whether those events improve performance similarly across perceptual tasks.

The role of sleep in speech-sound learning may be particularly relevant for populations characterized by differences in speech and language processing. Atypical sleep architecture has been reported in individuals with dyslexia (see Solbi and Earle, 2025, for a review) and autism spectrum disorder (Veatch et al., 2015). Although atypicality in the behavioral indices of consolidation has also been reported in developmental language disorder (DLD), these findings have not been explicitly linked to sleep processes (Earle et al., 2018; Earle and Ullman, 2021). DLD is a neurodevelopmental disorder that affects language acquisition in approximately 7% of children in the United States (Tomblin et al., 1997). Beyond language learning difficulties, individuals with DLD also experience deficits in speech perception that can further impact communication (Bishop and Snowling, 2004). Evidence of impaired overnight consolidation in speech-perceptual learning in this population (Earle et al., 2018) raises the possibility that sleep-dependent consolidation mechanisms may vary as a function of language ability (McGregor and Alper, 2015).

In the present study, we examined how sleep spindles and SOs relate to speech-sound memory consolidation in adults spanning a broad range of language ability, inclusive of individuals with typical language histories to those with DLD. We trained adults in the perception of a non-native contrast (dental-retroflex contrast in Hindi) in the context of minimally pairing “words,” mapping the target contrast to a set of visual objects. We measured their perceptual ability in the target sounds immediately after training and following a polysomnography-monitored nap. Our primary aim in this study was to investigate how individual differences in sleep physiology relate to consolidation trajectories across participants with varying language abilities as opposed to comparing consolidation processes in sleep vs. wake state. As such, we did not employ a wake-state control. We predicted that the post-training delay inclusive of a nap period would support improvements in task performance overall. In addition, we expected SO and spindle activity to differ according to language status, and perhaps as result, behavioral outcomes of consolidation. Specifically, we expected to observe smaller offline gains in adults with DLD compared to adults with typical language, consistent with prior reports of impaired overnight consolidation in this population (Earle et al., 2018). Furthermore, we predicted that language status would modulate SO and spindle activity, as well as SO-spindle coupling.

## 2 Methods

### 2.1 Materials

#### 2.1.1 Screening and classification materials

Assessments for language ability included the Modified Token Task and the Spelling task as described in Fidler et al. (2011, 2013). In addition, Word Attack (nonword) and Word Identification (real word) subtests of the Woodcock Reading Mastery Tests – Third edition (WRMT-III; Woodcock, 2011) and the Phonemic Decoding (nonword) and Sight Word Efficiency (real word) subtests of the Test of Word Reading Efficiency – Second edition (TOWRE-2; Torgesen et al., 2012) were administered. Hearing function was assessed using an Ambco 650A Audiometer (Ambco Electronics, Tustin, CA).

#### 2.1.2 Speech-perceptual training experiment

Stimulus presentation and response recording was controlled using E-Prime 3.0 (Psychology software tools, Inc.) on a Dell laptop equipped with high-quality circum-aural headphones. To avoid excessive light stimulation prior to a nap, a blue light filter was enabled (‘Night light’ feature in Windows 10) on our computers.

Auditory tokens (5 each of /ɖug/, /d̪ug/) produced by a male, native Hindi speaker were digitally recorded in a sound-proof audiology booth. Tokens were cut to the onset of the burst and rescaled to 70 dB SPL. Novel visual objects were obtained from the Tarrlab stimulus repository.^1^ Auditory tokens were presented at 70 dB SPL.

#### 2.1.3 Polysomnography recording and preprocessing of sleep

Sleep electroencephalogram (EEG), electrooculogram (EOG), and electromyogram (EMG) were conducted using an ActiChamp amplifier (Brain Products, GmbH) fitted with 32-channel actiCAP slim electrodes attached to a pre-positioned cap. Data acquisition and recording was controlled using BrainVision Recorder software (Brain Products, GmbH). The EEG data were preprocessed using the EEGLAB toolbox in MATLAB 2021 (Delorme and Makeig, 2004).

### 2.2 Participants

All participants provided informed consent according to procedures approved by the University of Delaware Institutional Review Board (IRB) prior to participation. Recruitment materials indicated that participants were required to be monolingual speakers of American English aged 18–35, with a history of typical gestation, hearing, and sensory-motor development, and no neurological or socio-emotional disorders. In addition to posting flyers, the study was advertised through the University of Delaware Disability Support Services (DSS) to recruit those with a history of language disorder. Individuals with a current diagnosis of attention deficit/hyperactivity disorder (ADHD) were excluded since ADHD medication can alter sleep (Owens, 2005). In addition, participants were required to pass a pure tone audiometric screening at 0.5-6 kHz administered at 25 dB at the time of the study. Participants were also screened for the presence of dyslexia (standard scores <85 on 2/4 measures of word-level reading) to ensure that performance on the DLD classification tasks (especially Spelling task, see below) could not be attributed a disorder of reading.

Participants were classified as DLD status if they reported a history of receiving services and obtained scores that resulted in a positive index for DLD under the methods devised by Fidler et al. (2011, 2013). Participants were assigned to the typically developed (TD) group if they indicated on their self-report that they had no history of speech, language, or reading remediation, and they obtained scores that resulted in a negative index for DLD using the same methods described below.

The Fidler et al. (2011, 2013) method utilizes raw scores of a 15-word spelling and modified token tests entered into the following equation Equation 1 (derived from the authors’ discriminant analysis):


y=6.5727+spelling∗−.2184+token∗−.1298 (1)

Individuals with y-values (Language Impairment [LI] index) above 0 are identified as positive for DLD, while individuals with y-values below 0 are negative for DLD. In the absence of standardized tools for identifying DLD in adulthood beyond age 22, the procedures described by Fidler et al. (2011, 2013) are widely accepted by the research community (e.g., Earle et al., 2018; Earle and Del Tufo, 2021; McGregor et al., 2017; Suddarth et al., 2012).

We had initially sought to recruit equal samples of adults with and without DLD into our study, however our data collection efforts were interrupted by the prolonged closure of our laboratory during the COVID-19 pandemic. Prior to lab closure, we enrolled a total of 35 participants into the study. 4 participants did not complete the study due to equipment failure, 1 dataset was lost to experimenter error, and 1 dataset was lost to software malfunction. Here, we report on the behavior of the remaining 29 participants (20 TD; 9 DLD; 25 White not Hispanic/Latinx, 1 White Hispanic/Latinx, 1 Multiracial, 2 Black). Of these, sleep data from 3 additional participants were excluded due to poor data quality. The demographic characteristics of our sample are presented in Table 1.

**Table 1:** Demographic characteristics of study participants.

Characteristic	TD (*n* = 20)	DLD (*n* = 9)
Age (years, *M ± SD*)	20.92 ± 2.00	20.34 ± 1.51
Sex (Female/Male)	15/5	8/1
Handedness (R/L)	19/1	8/1

Age is reported as mean (M) ± standard deviation (SD). Sex is reported as female/male, and handedness as right/left.

### 2.3 Procedures

#### 2.3.1 Study overview

In order to take advantage of the natural cosine rhythms of sleep propensity that peaks in the early afternoon for this age group (Lack and Lushington, 1996), all participants were scheduled from noon to 5 p.m. for a single study session. To promote sleep propensity, the laboratory was dimly lit with low-frequency (red) light for the duration of the pre-nap portion of the study. Please see Figure 1 for an overview of the study timeline.

**Figure 1:**
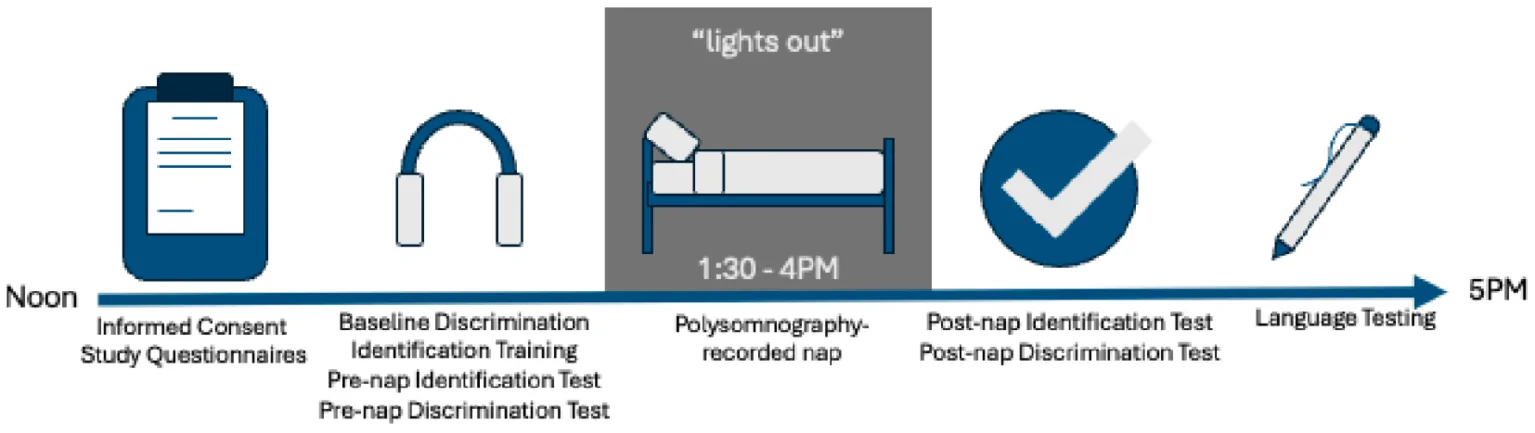
Overview of study design. Figure presents a timeline of study procedures for the 5-h study protocol. Participants arrived around noon to complete informed consent and a series of pre-nap tasks, with the goal of entering the “lights out period” at approximately 1:30 p.m., to last for 2.5 h. Around 4 p.m., participants were gently woken, and given approximately 15 min to get out of bed, remove the EEG cap and clean their hair, in order to overcome sleep inertia prior to engaging in post-nap tasks.

Upon enrollment, participants filled out an interview questionnaire asking for demographic information and factors that may influence outcome measures such as, language background, language and reading development, development of motor skills, and handedness (Modified Edinburgh Handedness Inventory; Veale, 2014). Participants also completed the Pittsburg Sleep Quality Index (Buysse et al., 1989), Epworth Sleepiness Scale (Johns, 1991), and the Horne-Ostberg Morningness-Eveningness Questionnaire (Adan and Almirall, 1991). They then completed a pure-tone audiometric screening (0.5-6 kHz presented at 25 dB) to ensure eligibility prior to starting the experimental tasks.

Once eligibility was confirmed, participants completed the nonnative speech training task. They were then fitted with the electrode cap and invited to take a nap while we recorded their neural activity using EEG. Participants underwent a “lights out” period of 2.5 h, after which they were gently awoken. The electrode cap was then removed, and participants were given a minimum of 15 min to overcome sleep inertia prior to completing the post-nap nonnative speech assessment. Participants were then administered the language and reading measures that were used to characterize our sample (Table 2).

**Table 2:** Language and reading performance by study participants.

Measure	TD	DLD	*p*-value
Modified token task	40.9 ± 3.52	32.0 ± 4.92	>0.001*
Spelling	11.35 ± 1.98	8.56 ± 2.19	0.003*
Word ID	42 ± 2.34	39.89 ± 2.71	0.022
Word attack raw	23.2 ± 2.33	20.0 ± 4.53	0.021
Sight Word Eff Raw	93.55 ± 9.41	89.44 ± 8.59	0.298
Phonemic decode raw	55.35 ± 5.25	47.44 ± 10.35	0.013

Values represent the mean score with standard deviation (SD) for each language measure. The Modified Token Task, Spelling, and Reading Task scores (Word Identification, Word Attack Raw, Sight Word Efficiency Raw, Phonemic Decoding Raw) were compared using a two-tailed T test between groups (TD vs. DLD). As expected for a DLD sample, these scores indicate group-level differences in language. *denotes statistical significance at 0.05 level after Bonferroni correction for multiple comparisons.

#### 2.3.2 Nonnative speech sound training and assessments

Participants were told that they would learn a new set of speech sounds through a series of tasks. A baseline discrimination task was administered first. During this task, participants were played two speech tokens (1 s inter-stimulus interval) and were asked to indicate if the word onsets belong to the same or different speech sound category. Each test contained 32 trials each of ‘Same’ and ‘Different’ pairs (64 total). ‘Different’ trials included dental and retroflex tokens (/ɖug/, /d̪ug/). ‘Same’ trials included two acoustically different tokens (e.g., /ɖug1/, /ɖug2/), such that judgments were based on category membership rather than acoustic similarity.

Participants were then exposed to a familiarization sequence in which the auditory token set was presented simultaneously with its corresponding novel visual object. Following this, participants completed 200 trials of 2-item, forced-choice identification training. Participants were asked to choose the image that corresponded to the token that they heard. Participants were presented with written feedback (“Correct!” vs. “Incorrect”) after each response.

Participants were assessed in their perception of the /ɖ/−/d̪/ contrast immediately after training, and again after a 3-h delay. The assessment included an identification test (50 trials of the training task without feedback) and a discrimination test. The discrimination tests were identical to the task administered at baseline.

Our performance measures of speech-perception ability were discrimination performance assessed at baseline (pre-training), and two time points after training: pre-nap and post-nap, and identification performance assessed at pre-nap and post-nap. As often utilized for speech perception tasks, D′ scores (*Signal Detection Theory*, Macmillan, 2002) were calculated for each time point by participant to track performance. D′ is a measure of perceptual sensitivity that accounts for the potential for response bias, expressed as the difference between Z-scores of hit and false alarm rates. A value of zero indicates chance performance and larger values indicate greater perceptual sensitivity.

#### 2.3.3 Polysomnography recording

Participants were seated comfortably in our sleep lab, dimly lit with low-frequency (red) light. In order to conserve time in electrode placement/application, participants were fitted with a pre-positioned 32-electrode cap, inclusive of the positions in the 10–20 system (Fp1, Fp2, F7, F3, F4, F8, FC5, FC1, FC6, M1, T7, C3, Cz, C4, T8, M2, CP2, P7, P3, Pz, P4, P8, O1, Oz, and O2). Two electro-oculographic (EOG) channels were placed above (right) and below (left) the eyes to monitor eye movements, and three electromyography (EMG) channels placed on the chin monitored muscle tone. Electrolytic gel was applied between electrode tips and scalp to optimize impedance to <25 kOhms prior to recording.

After minimizing impedance and calibration, participants were asked to lie down comfortably on the bed. Participants were asked to close their eyes and rest/sleep to the best of their ability. Participants were advised that they could communicate with the experimenters if needed by speaking into a baby monitor placed in the nap room. Lights were turned out, and the experimenter exited to the adjacent room. PSG was recorded continuously throughout the rest period, sampling at 500 Hz.

Using MATLAB (R2021a), a band pass filter between 0.1 and 50 Hz was applied to remove low- and high-frequency artifacts from the EEG signal. Additionally, movement artifacts were removed manually through visual inspection. Data from two participants were excluded due to high noise levels. The data were scored into stages 1, 2, slow-wave sleep (SWS), and REM sleep using an automated sleep scoring software implemented in Python, as described by Vallat and Walker (2021).

#### 2.3.4 SO and spindle identification

The EEG signal was low pass filtered at 3.5 Hz. Next, the positive to negative zero-crossing were registered and the consecutive ones within an interval 0.8–2 s were selected. The negative peak of SOs was determined if the amplitude of the negative peak was larger than 1.25 times the averaged negative peak. Finally, the amplitude difference between the negative and positive peaks had to be larger than 1.25 times the averaged difference between the negative and positive peaks (Delorme and Makeig, 2004; Dehnavi et al., 2023).

The EEG data were band-pass filtered with FIR filters between 9–12 and 13–16 Hz for detecting slow and fast spindles, respectively. Next, the root mean square (RMS) of the filtered signals was calculated at every point using a moving window of 0.2 s and smoothed using a moving window of length 0.2 s. After computing the standard deviation (STD) of the filtered signals, a threshold was defined as 1.5 × STD. A spindle was detected if the RMS signal remained above the defined threshold for 0.5–3 s. Once the spindles were detected the time point of the spindle event was defined as the time of maximum peak (Mölle et al., 2011; Klinzing et al., 2016).

Slow and fast spindles were quantified within *a priori* regions of interest (ROIs) based on their characteristic scalp topographies. Slow spindles demonstrate a predominantly anterior distribution, whereas fast spindles are expressed strongly over central and parietal regions (Werth et al., 1997; Zeitlhofer et al., 1997; Cox et al., 2017). Accordingly, average slow spindle density was calculated across F3 and F4, and average fast spindle density was calculated across C3 and C4 (Figure 2). These channels were selected as representative, matched samples of the established anterior vs. central distributions, while excluding the midline channels in order to avoid the topographical overlap between slow and fast spindles (Zeitlhofer et al., 1997). As only nine participants reached SWS sleep during their nap, SO density was examined across NREM sleep inclusive of stage 2.

**Figure 2:**
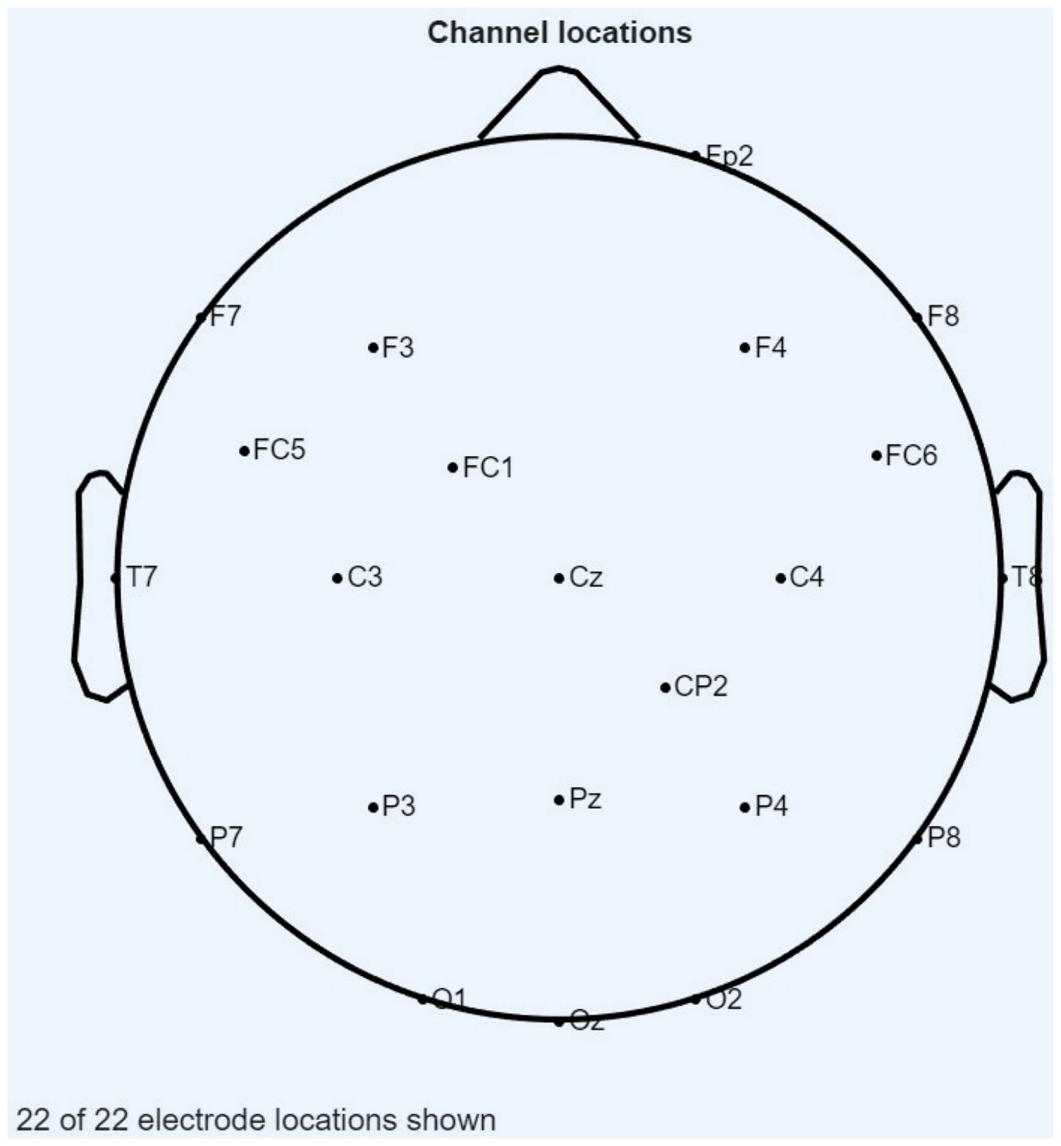
Electrode montage. The 22 scalp channels retained for analysis, with channel labels. Frontal region of interest: F3, F4. Central region of interest: C3, C4.

#### 2.3.5 SO-spindle coupling

The temporal relationship between spindles and the SO cycle, rather than the density of either event alone, is increasingly held to be the mechanistically specific marker of consolidation (Helfrich et al., 2018; Denis et al., 2021; Lutz et al., 2026). To examine the timing of sleep spindles relative to the SO cycle, SO-spindle coupling was estimated in the same ROIs using the continuous synchronization index described by Ladenbauer et al. (2017). As not all participants entered SWS during their “lights out” period, coupling estimates were restricted to stage 2 within NREM sleep. Recordings were down-sampled to 250 Hz, and slow oscillations were detected by band-pass filtering at 0.16–1.25 Hz, identifying negative half-waves lasting between 0.8 and 2.0 s, and retaining the largest 25 percent by peak-to-peak amplitude. The instantaneous SO phase was obtained by filtering the same Channels were filtered at 0.5–1.25 Hz and applying the Hilbert transform, with zero degrees corresponding to the SO up-state and 180 degrees to the trough.

Spindle activity was quantified as the squared amplitude envelope of the signal band-pass filtered at 9–12 Hz for slow spindles and at 13–16 Hz for fast spindles. The mean-removed envelope was itself filtered in the SO band and Hilbert-transformed, so that the phase of the spindle power fluctuation could be examined directly against the phase of the SO. For each detected slow oscillation, the synchronization index (SI) was computed as the mean complex phase difference across a 2-s window centered on the trough. The angle of the mean SI across all events and channels within the region was calculated for each participant.

### 2.4 Statistical analyses

All analyses were conducted in either MATLAB (R2021a) or R (version 4.6.0) and R Studio (version 2026.05.1 + 225; R Core Team, 2026). Preprocessed data and analysis code is available through the Open Science Framework.^2^

We note that we conduct several group-level (TD vs. DLD) comparisons that we had planned before the pandemic-related disruption to our recruitment efforts, with the acknowledgement that the small size of the DLD sample is a substantial limitation to interpreting these results. We therefore follow up on our group-level comparisons with correlational analyses that treat the LI index, that is, our measure of difficulties with language, as a continuous variable, to ensure that we are fully capturing the insights offered by this dataset.

#### 2.4.1 Behavioral analyses

To model for the repeated measurements in perceptual ability, we employed a linear mixed-effects (LME) approach (Baayen et al., 2008), on the basis that mixed-effects approaches are more robust to unequal groups relative to analyses of variance (Heo and Leon, 2005; Schielzeth et al., 2020). To determine whether perceptual ability improved through training, discrimination performance was modeled with Time (pre-training vs. pre-nap), Group (DLD vs. TD), and their interaction (Time × Group) as fixed effects, and by-participant intercepts modeled as a random effect. To determine whether perceptual ability improved after sleep, we modeled for discrimination and identification scores separately, each model with Time (pre-nap vs. post-nap), Group (DLD vs. TD), and their interaction (Time × Group) as fixed effects, baseline discrimination performance as covariate, and by-participant intercepts modeled as a random effect.

#### 2.4.2 Comparisons of sleep characteristics across DLD and TD

To investigate if sleep characteristics differed as a function of Group (DLD vs. TD), we conducted 2-tailed, independent samples t-tests on the sleep stage durations (stage 2, SWS, and REM). Next, we examined potential differences between TD and DLD on sleep parameters that have been associated with memory consolidation in the learning and memory literature, namely spindle and SO densities. As the small sample of DLD may obscure potential group-level differences between language ability and sleep physiology, relationships between spindle and SO densities and the LI index also were assessed using Spearman’s rank-order correlations, with the Benjamini-Hochberg adjustment applied to correct for false discovery rate (FDR).

To determine if there were any group-level differences in the temporal relationship between spindle activity and SO cycles, two properties of SO-spindle coupling were examined. Preferred phase, defined as the point in the SO cycle at which spindle power was maximal, was evaluated at the group level using the Rayleigh test of nonuniformity. The group mean angle was interpreted only when the resultant vector length exceeded the chance-based critical value given the sample size. If the angle did not meet this threshold, the angle and resultant vector length were reported descriptively without group-level comparison. Statistical tests of group differences were carried out by 2-tailed, independent samples *t*-tests.

As an exploratory set of analyses, we examined potential differences in scalp distributions of spindle and SO densities by examining channel-wise parameter estimates for the effect of group (TD vs. DLD). Please see Supplementary materials for the results on these analyses.

#### 2.4.3 Relationships between sleep characteristics and behavior

To determine if behavioral change was related to spindle activity, post-nap perceptual behavior was modeled with baseline discrimination and pre-nap behavior, and an interaction term between spindle density and group (TD vs. DLD), as predictors. As with the LI index above, we followed up on these regression analyses with correlational analyses between sleep parameters and change scores (post-nap – pre-nap) to better capture the nuances of our dataset, with the Benjamini-Hochberg adjustment applied to control for FDR.

To determine if indexes of SO–spindle coupling was associated with behavior, we examined subject-level coupling estimates obtained from the Ladenbauer analysis. For each participant, coupling strength was quantified as the magnitude of the synchronization index (∣SI∣), and the corresponding SI angle represented the preferred phase for SO–spindle coupling. Coupling estimates were analyzed for slow and fast spindles separately and examined against changes in discrimination and identification performance, and also against our measure of language difficulty (LI index). Associations between coupling strength and behavioral measures were assessed using Spearman rank-order correlations (r). Associations between preferred coupling phase and behavioral measures were assessed using circular–linear correlations (rcl), which account for the circular nature of phase measurements. Correlations were conducted for the TD and DLD groups as well as for the pooled sample. Benjamini–Hochberg FDR correction was applied across the 36 correlations. For visualization, linear fits were used for coupling-strength associations, whereas quadratic fits were used to illustrate the relationship between coupling phase and behavioral measures.

## 3 Results

### 3.1 Behavioral results

#### 3.1.1 Discrimination performance

The initial LME model comparing baseline to pre-nap performance revealed a significant main effect of Time (*β* = 0.66, *SE* = 0.17, *t* = 3.81, *p* < 0.001), indicating a significant improvement in discrimination performance following training. However, there was no significant main effect of Group (*β* = −0.32, *SE* = 0.37, *t* = −0.87, *p* = 0.389), and no significant Time × Group interaction (*β* = −0.09, *SE* = 0.31, *t* = −0.27, *p* = 0.785), suggesting that overall discrimination performance and the magnitude of training-related improvement did not significantly differ between the DLD and TD groups.

When comparing pre-nap to post-nap performance, the LME model did not reveal any significant effects. The main effect of Time was not significant (*β* = 0.05, *SE* = 0.16, *t* = 0.31, *p* = 0.756), indicating that, on average, there was not a significant change in discrimination performance across the nap interval. Similarly, no significant main effect of Group was observed (*β* = −0.41, *SE* = 0.41, *t* = −0.98, *p* = 0.333), nor was there a significant Time × Group interaction (*β* = 0.24, *SE* = 0.30, *t* = 0.80, *p* = 0.429). These results suggest that changes in discrimination across the nap period were relatively subtle, and did not significantly differ between the DLD and TD groups (Table 3, Figure 3A).

**Table 3:** Discrimination performance over time.

Model	Effect	Estimate (β)	SE	t	DF	*p*	95% CI
Baseline → PreNap	Intercept	0.77	0.20	3.76	54	< 0.001	[0.36, 1.18]
	Time_PreNap	0.66	0.17	3.81	54	< 0.001	[0.31, 1.01]
	Group_DLD	−0.32	0.37	−0.87	54	0.389	[−1.06, 0.42]
	Time_PreNap × Group_DLD	−0.09	0.31	−0.27	54	0.785	[−0.71, 0.54]
PreNap → PostNap	Intercept	1.43	0.23	6.21	52	< 0.001	[0.97, 1.90]
	Time_PostNap	0.05	0.16	0.31	52	0.756	[−0.27, 0.38]
	Group_DLD	−0.41	0.41	−0.98	52	0.333	[−1.24, 0.43]
	Time_PostNap × Group_DLD	0.24	0.30	0.80	52	0.429	[−0.36, 0.83]

Fixed effects coefficients from the linear mixed-effects models assessing discrimination performance. The first model examined changes from baseline to pre-nap performance, while the second model examined changes from pre-nap to post-nap performance.

**Figure 3:**
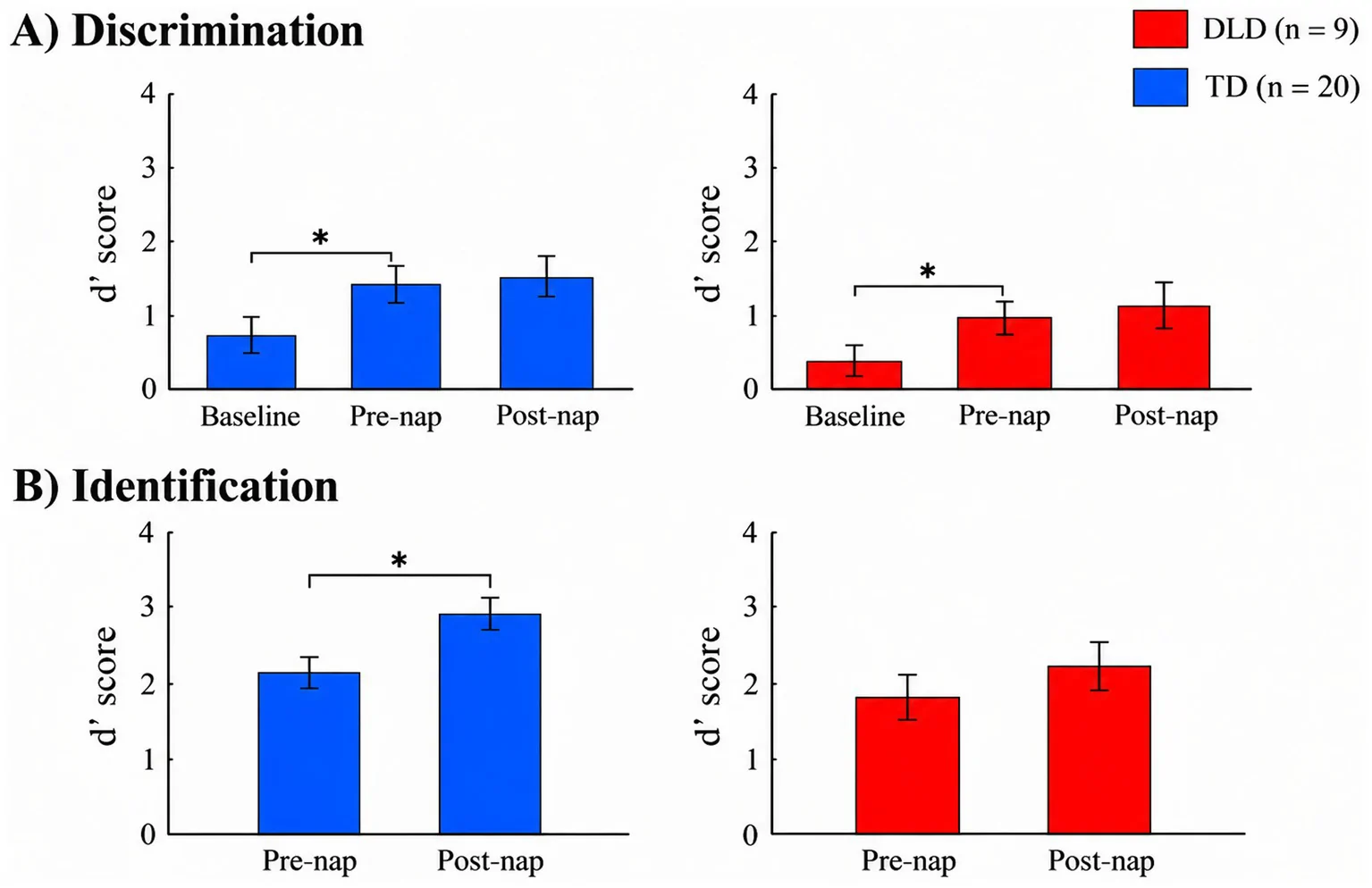
Changes in perceptual behavior over training and time. Mean d’ scores for the Discrimination **(A)** and Identification **(B)** tasks are displayed for TD (*n* = 20) and DLD (*n* = 9) groups across baseline, pre-nap (post-training), and post-nap time points. Panel A illustrates Discrimination performance, where the linear mixed effects (LME) model indicated a significant improvement from baseline to pre-nap (post-training) for both groups. Post-nap discrimination performance did not show significant changes, nor were there significant group effects or interactions. Panel B shows a significant improvement in Identification performance post-nap for the TD group, while no significant changes were observed for the DLD group. We note however that the LME model did not identify a significant interaction between changes to identification performance and group, suggesting that the magnitude of changes did not differ substantially between groups. Error bars represent the standard error of the mean (SEM).

#### 3.1.2 Identification performance

The LME model comparing pre-nap to post-nap identification performance revealed a significant main effect of Time (*β* = 0.61, *SE* = 0.26, *t* = 2.36, *p* = 0.022), indicating that identification performance significantly improved across the nap interval. However, no significant main effect of Group was observed (*β* = −0.61, *SE* = 0.56, *t* = −1.08, *p* = 0.284), nor was there a significant Time × Group interaction (*β* = −0.09, *SE* = 0.48, *t* = −0.19, *p* = 0.854). These findings suggest that while identification performance improved overall following the nap interval, the magnitude of improvement did not significantly differ between the DLD and TD groups (Table 4, Figure 3B).

**Table 4:** Identification performance over time.

Effect	Estimate (β)	SE	t	DF	*p*	95% CI
Intercept	2.15	0.31	6.85	52	< 0.001	[1.52, 2.77]
Time_PostNap	0.61	0.26	2.36	52	0.022	[0.09, 1.14]
Group_DLD	−0.61	0.56	−1.08	52	0.284	[−1.74, 0.52]
Time_PostNap × Group_DLD	−0.09	0.48	−0.19	52	0.854	[−1.04, 0.87]

Fixed effects coefficients from the linear mixed-effects model assessing identification performance from pre-nap to post-nap.

### 3.2 Sleep characteristics

For sleep stages, the comparison of sleep stages duration between the TD and DLD groups revealed no statistically significant differences. For stage 2 (S2) sleep, the mean duration was 35 min in the TD group and 36.5 min in the DLD group, with an estimated difference of 1.5 min (*SE =* 7.24*, p* = 0.837). Slow-wave sleep (SWS) duration was slightly longer in the TD group (6.13 min) compared to the DLD group (2 min), but this difference was not statistically significant (*β =* −4.13*, SE =* 3.15*, p =* 0.201). REM sleep duration was also comparable between the two groups, with means of 20.34 min for TD group and 18.75 min for DLD (*β =* −1.59*, SE =* 4.97*, p =* 0.751).

For the sleep parameters that have been associated with memory consolidation in the learning and memory literature, differences between groups were not statistically significant, although average slow sleep spindle density was numerically lower in those with DLD relative to TD (*p* = 0.067). These values furthermore were not significantly associated with the LI index. Please see Table 5 for a descriptive summary of sleep characteristics across groups.

**Table 5:** Comparison of sleep characteristics in across groups.

Sleep characteristic	Mean		tStat	*p*-value
	TD	DLD		
Duration (mins)				
S2	35.00	36.50	0.21	0.837
SWS	6.13	2.00	−1.31	0.201
REM	20.34	18.75	−0.32	0.751
Density				
Slow spindle	3.50	2.95	1.92	0.067
Fast spindle	4.40	4.80	−1.08	0.289
SO	5.09	5.09	<0.01	0.999

Table presents durations of stage 2 sleep (S2), slow-wave sleep (SWS), and REM sleep for the TD and DLD groups, as well as slow and fast spindle density and slow oscillation (SO) density. No statistically significant differences were observed between the groups.

For SO-spindle coupling, slow spindles demonstrated a consistent, preferred SO phase in the TD group (*p* = 0.006), and uniformity of phase distribution fell just short of significance for the DLD group (*p* = 0.051). For both groups, the coupling angles were concentrated around the SO trough (Figure 4). In contrast, fast-spindle coupling angles were not significantly concentrated in either group, providing no evidence of a common preferred coupling phase. Group-level comparisons were therefore carried out only for slow spindle-SO coupling, and neither phase angle nor coupling strength were found to significantly differ across groups. See Table 6 for descriptive summary.

**Figure 4:**
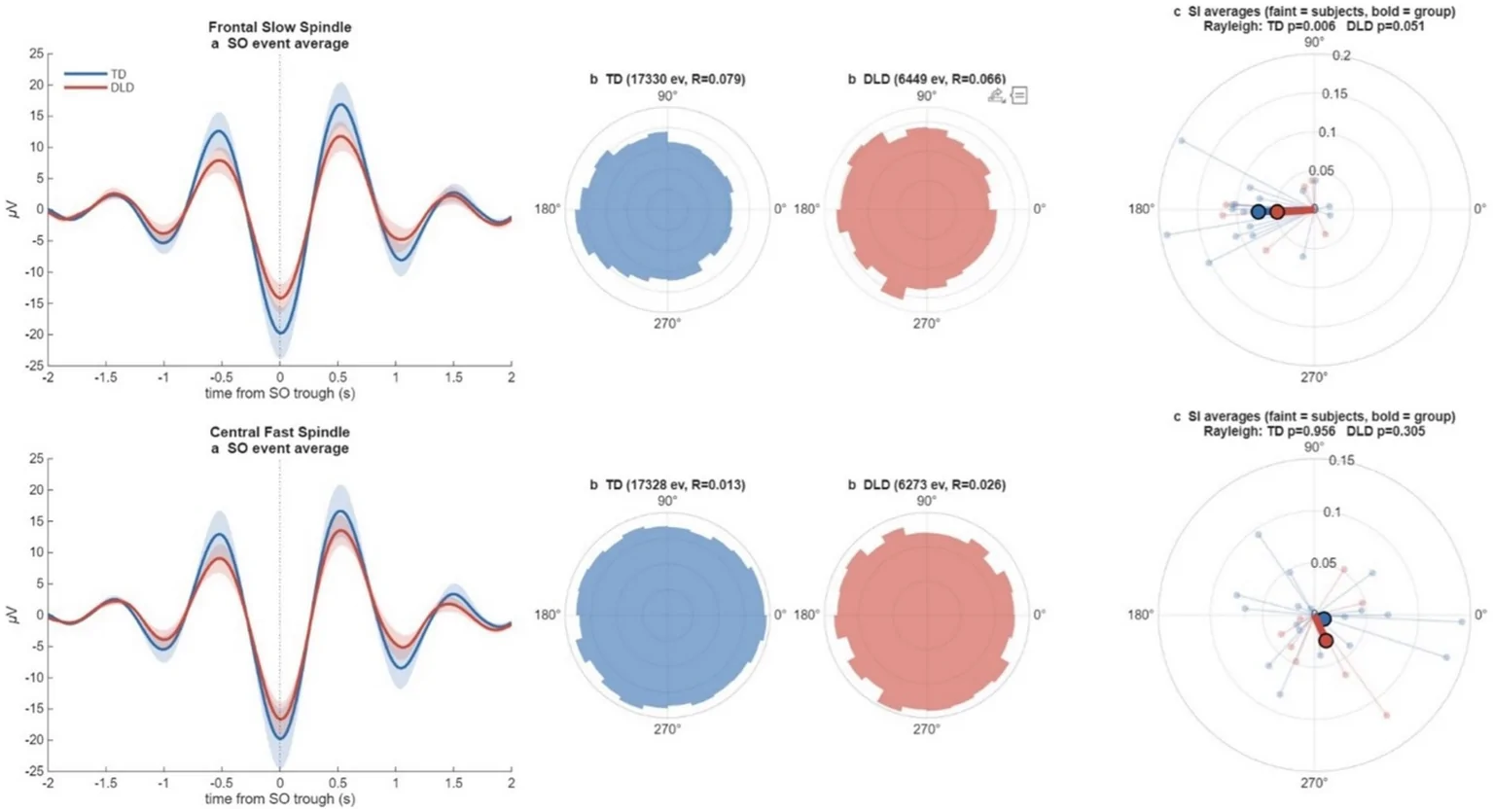
SO-spindle coupling by group. Coupling between slow oscillations (SOs) and spindle power. SO-locked coupling for frontal slow spindles (F3, F4; top row) and central fast spindles (C3, C4; bottom row) in TD (blue) and DLD (red) participants. **(A)** The left panel displays group-averaged SO waveforms time-locked to the SO trough (0 s); shaded regions indicate ±SEM. **(B)** The center panels display SI phase distributions based on all detected SO events within each group; *n* denotes total detected SO events. **(C)** The right panel displays participant-level SI vectors, where angle represents preferred phase and vector length represents coupling strength (∣SI∣); faint vectors indicate individual participants and bold vectors indicate group means. Phase is referenced to the SO, with 0° representing the up-state and ±180° representing the trough. Rayleigh tests of participant-level preferred phase are shown for each group.

**Table 6:** Group descriptives on SO-spindle coupling.

So-spindle coupling metric	Group	N	Mean	SD	SE	Coefficient of variation
Slow spindle SI strength	DLD	8	0.06	0.035	0.01	0.56
	TD	18	0.09	0.054	0.01	0.63
Slow spindle distance from upstate (degrees)	DLD	8	139.4	43.78	15.48	0.31
	TD	18	131.3	58.50	13.79	0.45
N slow spindle events	DLD	8	806.1	488.6	172.7	0.61
	TD	18	962.8	520.7	122.7	0.54
Fast spindle SI strength	DLD	8	0.05	0.03	0.01	0.58
	TD	18	0.06	0.04	0.01	0.63
Fast spindle distance from upstate (degrees)	DLD	8	92.63	53.28	18.84	0.58
	TD	18	87.65	63.62	15.00	0.73
N fast spindle events	DLD	8	784.1	478.7	169.3	0.61
	TD	18	962.7	522.6	123.2	0.54

Table presents means and distributions (standard deviation [SD] and standard error [SE]) of SO-spindle coupling metrics by Group. No statistically significant differences were observed between the groups.

Taken together, these results suggest that sleep duration, ROI-constrained measures of spindle and SO densities, nor SO-spindle coupling, do not meaningfully differ across language status. This observation warrants the caveat however that our sample of adults of DLD is small. Furthermore, our exploratory analyses suggest potential differences in the scalp topography of fast spindle activity between adults with and without DLD. Please see Supplementary materials for comparisons between DLD and TD on scalp distributions for slow and fast spindle density, and SO density.

### 3.3 Relationships between sleep characteristics and behavior

#### 3.3.1 Relationships between spindle and SO density and discrimination performance

After controlling for baseline and pre-nap discrimination performance, greater slow spindle density significantly predicted better post-nap discrimination performance (*EST* = 0.45, *SE* = 0.21, *t* = 2.13, *p* = 0.046), whereas greater SO density significantly predicted poorer post-nap performance (*EST* = −0.43, *SE* = 0.15, *t* = −2.94, *p* = 0.008). Fast spindle density was not significantly associated with post-nap discrimination performance. Neither the association with slow spindle density nor the association with SO density were moderated by language status. See Figure 4 for effect plots of slow spindle and SO density on post-nap discrimination scores.

Correlational analyses between sleep parameters and Δdiscrimination and Δidentification confirmed the directionality of these associations, that is, fast spindle and SO densities appeared, in general, to be negatively correlated with changes in perceptual behavior (especially in the TD cohort), while slow spindle density appeared to have a positive relationship with changes to behavior. It is important to note however that none of these correlations were statistically significant after the Benjamini-Hochberg adjustment was applied to control for FDR for 27 comparisons. See Figure 5 for a summary depiction of correlations.

**Figure 5:**
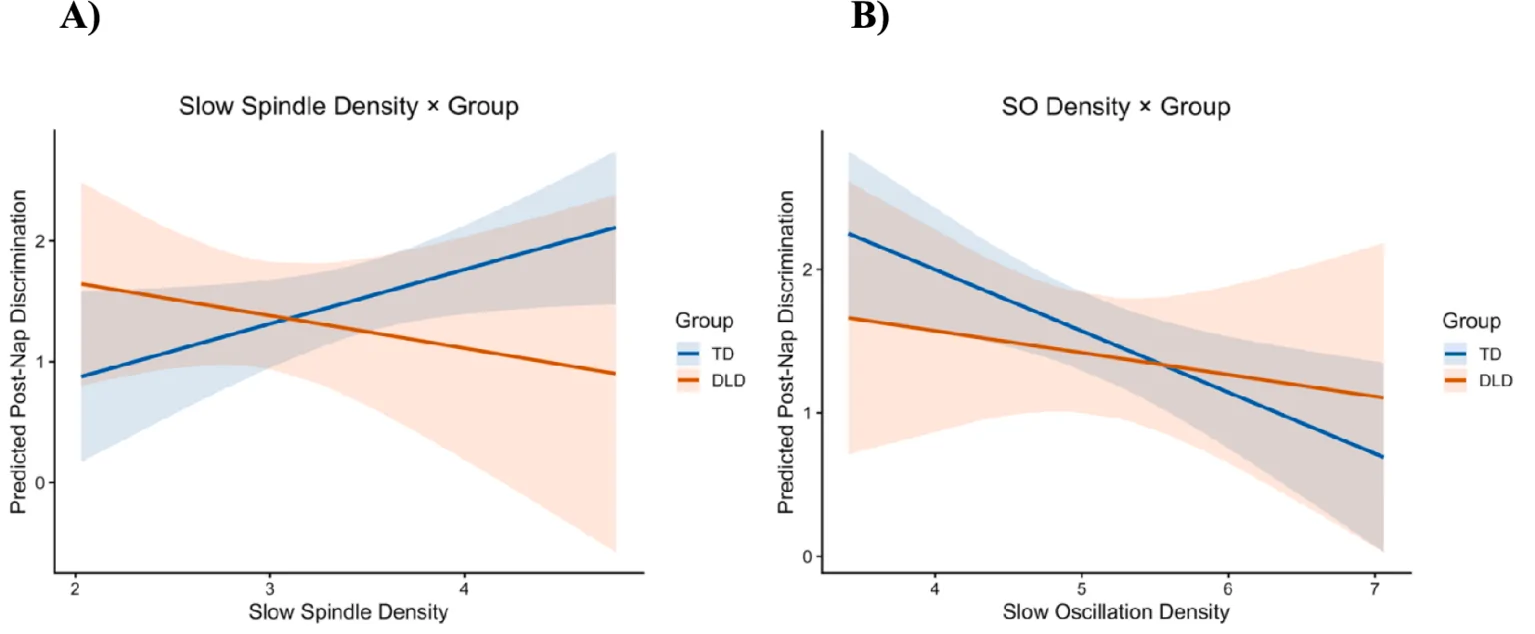
Effect plots for the predictive value of slow spindle and SO density on perceptual discrimination. Figure presents the predictive effect of the **(A)** slow spindle density and **(B)** slow oscillation (SO) density by Group, on the predicted performance on the post-nap discrimination task (D′ score) after controlling for baseline and pre-nap performance. Shaded regions represent the 95% confidence intervals of the estimate.

#### 3.3.2 Relationships between spindle and SO density and identification performance

After controlling for baseline and pre-nap identification performance, post-nap identification performance was not significantly predicted by spindle density, nor by SO density. At face value, this may indicate that changes to identification performance was not related to consolidation processes during sleep. However, we note that the r-value of the relationship between fast spindle density and Δidentification for TD adults was −0.52 (see Figure 6). Thus, a potential relationship between fast spindle density and changes to perceptual identification behavior may have been obscured by the relatively low power afforded by the current sample size.

**Figure 6:**
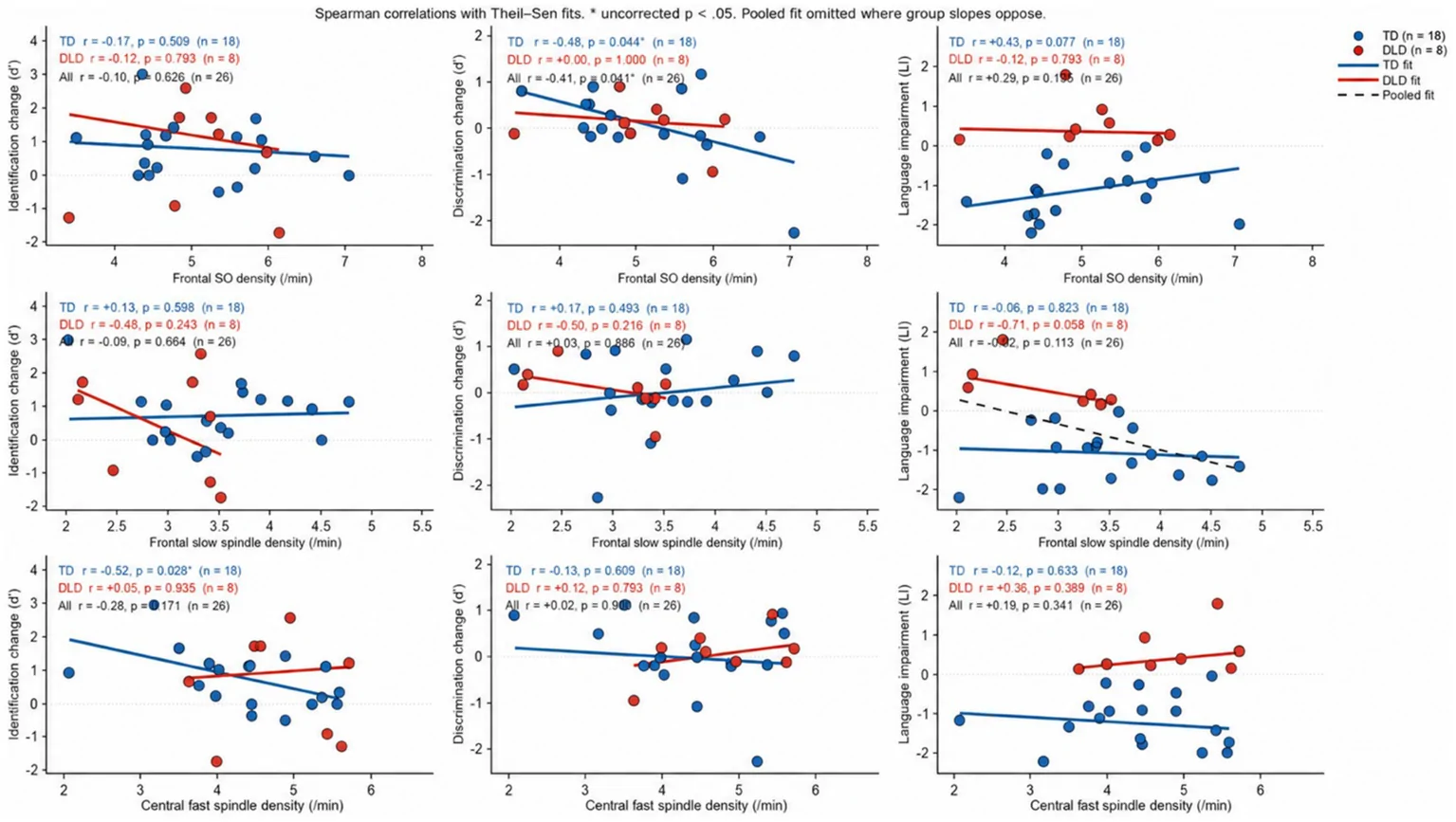
Correlations between SO and spindle density and behavioral tasks. Correlations across SO density, slow spindle density, and fast spindle density and behavioral outcomes. The figure presents uncorrected *p*-values. None of these correlations were statistically significant after the Benjamini-Hochberg adjustment was applied to control for false discovery rate.

#### 3.3.3 Relationships between SO-spindle coupling and behavior

For coupling metrics between slow spindles and SO, neither coupling strength nor the preferred phase angle were significantly correlated with changes in perceptual behavior after the Benjamini-Hochberg adjustment for FDR. Coupling strength was significantly correlated with the LI index in a negative direction for the DLD group (*r =* −0.71, *p =* 0.047), indicating that those with higher coupling strength were found to have lesser difficulties with language. See Figure 7 for summary of relationships between SO-slow spindle coupling metrics and behavior.

**Figure 7:**
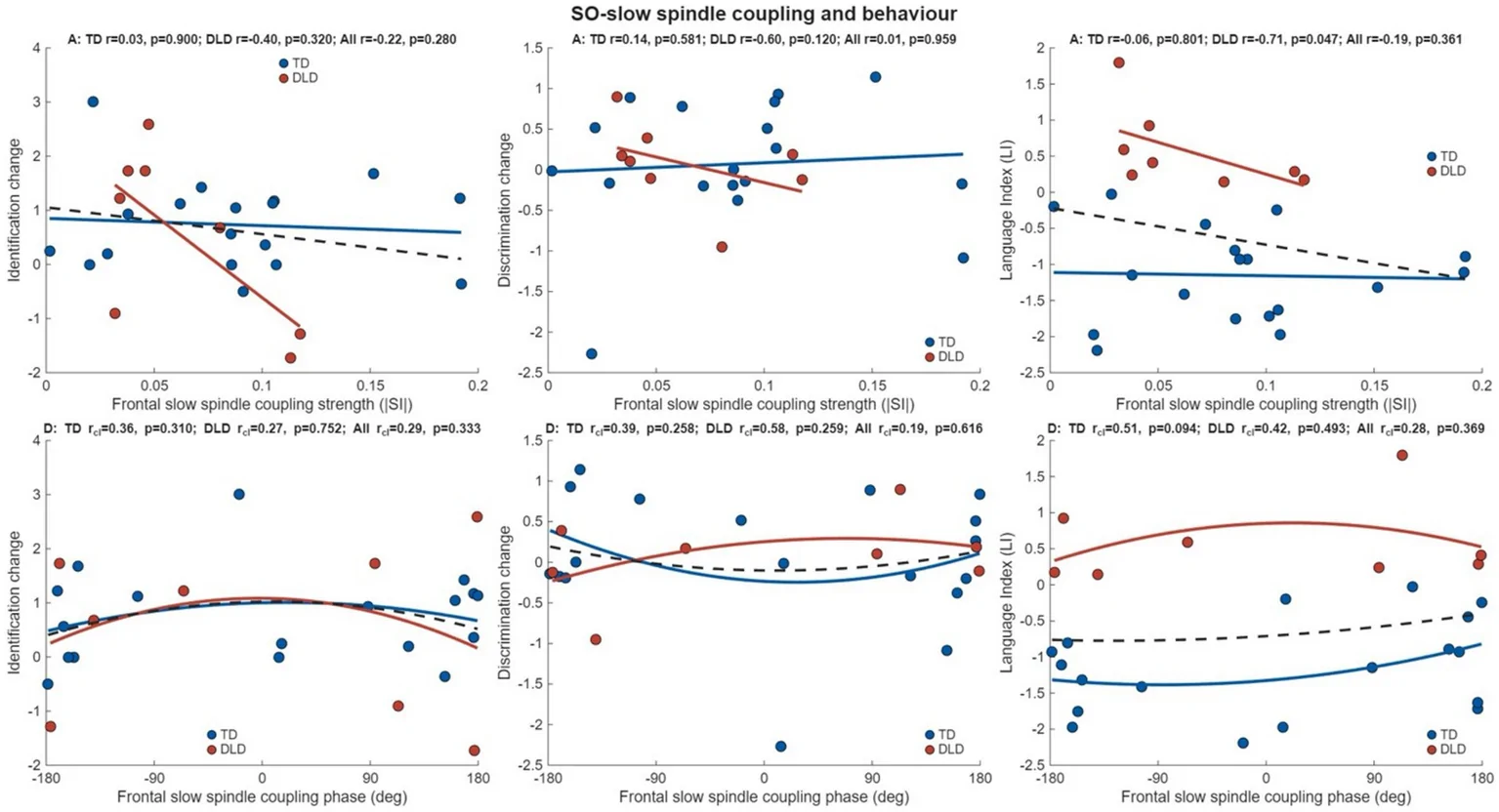
SO-slow spindle coupling and behavior. Correlations across slow spindle coupling strength (|SI|), coupling phase angle, and behavioral outcomes. *P*-values are adjusted for the Benjamini-Hochberg correction to control for false discovery rate.

For coupling metrics between fast spindles and SO, none of the relationships were statistically significant after applying the Benjamini-Hochberg adjustment to *p*-values to control for FDR (Figure 8). We note that the relatively high r values for coupling strength and r_cr_ values for coupling phase were observed both with changes in identification and discrimination. Thus, the absence of significant correlations in the present dataset does not preclude the possibility that these relationships may emerge in a larger dataset.

**Figure 8:**
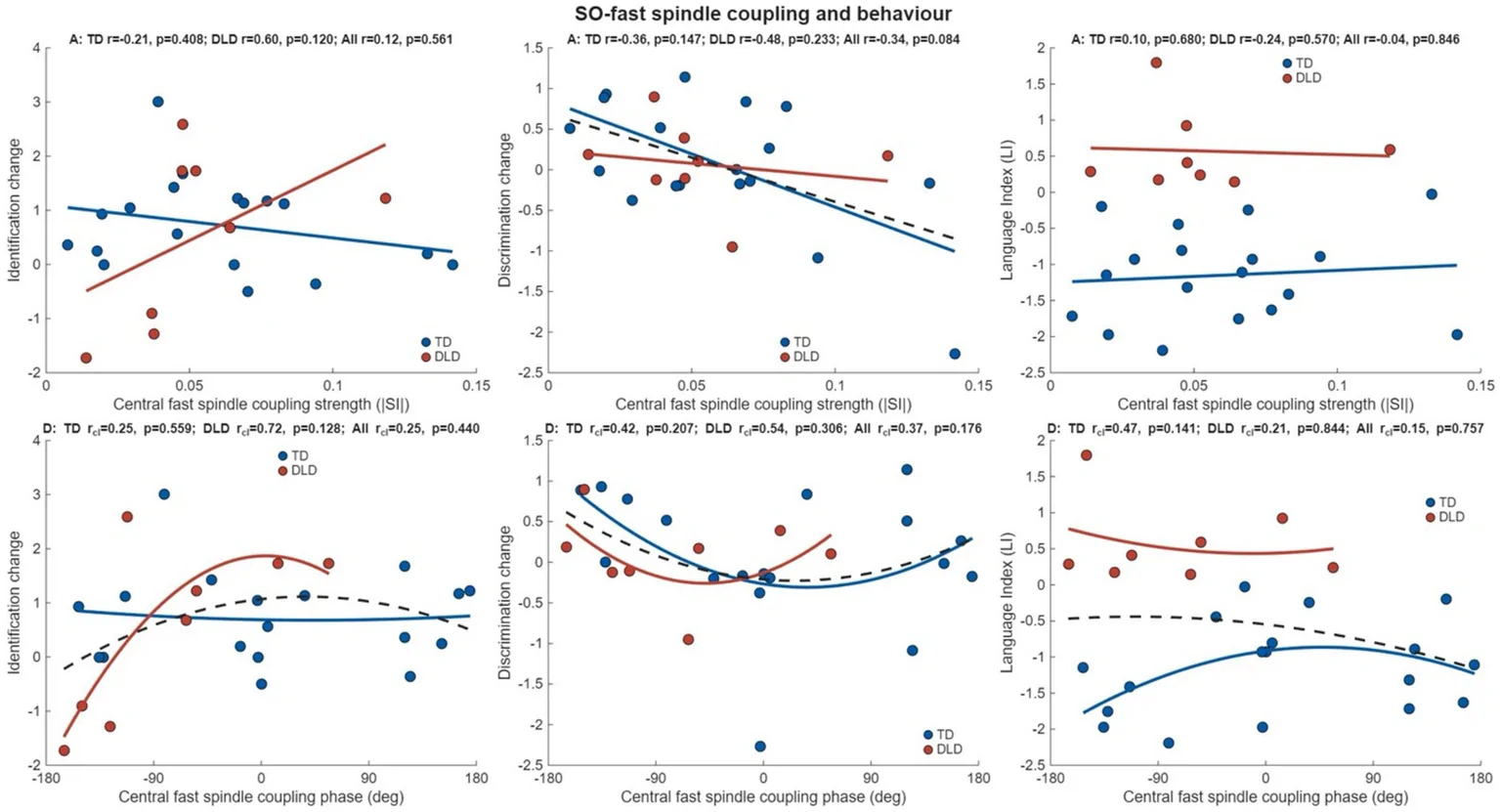
SO-fast spindle coupling and behavior. Correlations across fast spindle coupling strength (|SI|), coupling phase angle, and behavioral outcomes. *P*-values are adjusted for the Benjamini-Hochberg correction to control for false discovery rate.

## 4 Discussion

The current study investigated the role of a post-training nap in changes to speech-perceptual behavior following training on a difficult-to-perceive nonnative contrast. Our participants ranged in language ability, from those with typical language to those with a history of DLD. Below, we summarize the current findings in the context of the previous literature.

### 4.1 Behavioral findings

Our behavioral findings indicate that while participants’ perceptual identification ability improved over the nap period, a significant improvement was not observed at group level for the discrimination task. This is consistent with prior studies on the overnight consolidation of the dental/retroflex contrast, that have also consistently reported offline gains in identification ability, accompanied by subtle/non-significant changes to discrimination (Earle and Myers, 2015a, 2015b). While identification involves the recall of the trained token-label mapping, speech discrimination requires the spectrotemporal comparison of the acoustic signal. Thus, the two reflect distinct perceptual skills and the time course of changes to behavior may likewise differ.

The magnitude of offline changes to perceptual behavior was not modulated by language status. This observation differs from a previous account that reported a significant difference between TD and DLD adults in perceptual performance following overnight sleep, despite comparable training-induced gains observed in the evening prior (Earle et al., 2018). One possibility is that a nap is too short a duration of sleep for such group-level observations to emerge. Another may be that we were underpowered in our DLD sample to detect a significant interaction in our model. Indeed, an exploratory paired t-test conducted on identification scores by group suggests that whereas the TD participants improved, the DLD participants did not.

### 4.2 Comparisons of sleep characteristics across language status

The sleep characteristics of individuals with DLD has been a topic of interest in the literature for about a decade (Carranza Amores et al., 2017; Dlouha et al., 2020), prompted by associations between subjective reports of habitual sleep quality and language (Knowland et al., 2021; McGregor and Alper, 2015). In the current paper, we did not find any group-level differences in sleep duration.

We observed a numerically lower slow spindle density in adults with DLD relative to TD (*p* = 0.067). While we did not observe a difference in our ROI-based quantification of fast spindle density, our exploratory analyses suggest a potential difference between TD and DLD in scalp topography for fast spindles (see Supplementary materials). Moreover, we found that elevated spindle density in posterior regions was associated with greater difficulties with language. Interestingly, prior reports of spindle activity in populations with learning disabilities have suggested that children with Dyslexia have a higher sleep spindle density relative to TD children (Bruni et al., 2009; see Solbi and Earle, 2025, for review). Similarly, a collection of older studies identified increased spindle activity in children with learning disabilities, broadly defined (Bixler and Rhodes, 1968; Gibbs and Gibbs, 1962). These studies did not examine spindle topography however, thus it is unclear if the prior studies also found the elevations in spindle activity in disordered populations to be concentrated in posterior regions. Fogel et al. (2010) have suggested that higher baseline (i.e., non-learning related) spindle activity disrupts subsequent encoding and thus may reflect a maladaptive trait associated with poor cognitive performance. This dataset does not include a baseline polysomnographic measure of sleep. We are therefore unable to determine if adults with DLD exhibit elevated spindle activity relative to typical adults during habitual sleep, or if the increased spindle activity observed here is in response to learning, such as an elevated effort during the training task in DLD relative to TD. Nevertheless, these findings suggest a need for further research into spindle density in DLD, with larger samples and longer sleep duration.

### 4.3 Correlations between sleep characteristics and behavior

Previous research has generally demonstrated a positive relationship between sleep spindle density and declarative memory consolidation (see Fogel and Smith, 2011, for review). To our knowledge, the only prior work on the relationship between electrophysiological indexes of sleep and speech-perceptual learning is a recent investigation by Qin et al. (2025) on the learning of Cantonese tones. Here, the authors also found a positive relationship between generalization of learned tone contours and both fast spindles and SO. Here, we found that slow and fast spindle densities were associated with behavioral change in opposite directions, with the caveat that the correlation with fast spindle density was not statistically significant after FDR correction. Specifically, higher slow spindle density was positively associated with residual post-nap discrimination behavior, whereas fast spindle density was negatively correlated, albeit non-significantly, with changes to identification behavior. A similar directional pattern has previously been reported in Lustenberger et al. (2015), who reported a positive association between slow spindle density and retention of learned word pairs, but a negative association between retention and fast spindle density. The functional significance of these opposing associations is uncertain. Slow and fast spindles differ in topography, temporal coordination with the slow oscillation, and distribution across NREM sleep stages (Dehnavi et al., 2019). This may be particularly relevant in the current study, in which only a subset of participants reached SWS.

Surprisingly, we also observed a negative correlation between SO density and changes in perceptual discrimination performance. Again, the functional significance of the negative association is uncertain. NREM sleep characterized by a high density of SOs is thought to support systems-level reorganization, abstraction, and integration. For speech categories, that process may preserve category-level information while reducing access to token-specific acoustic detail. Higher SO density could therefore accompany stronger abstraction but poorer performance on a task requiring fine-grained acoustic comparison. This is consistent with recent observations that SWS promotes the preservation of category-level representations, at the expense of item-level information (Liu et al., 2025). An alternative possibility is that more frequent SOs could include many uncoupled events with poorly timed spindle activity, however we did not find any SO-spindle coupling metrics to be significantly correlated with changes to perceptual behavior after FDR correction.

Interestingly, we found the coupling strength of SO-slow spindle coupling to be negatively associated with language difficulties in those with DLD. In other words, those with stronger SO-spindle coupling had better language skills within the DLD sample. Given the small number of participants with DLD, this finding should be interpreted cautiously. Nevertheless, it raises the intriguing possibility that SO–spindle coupling may serve a compensatory or protective role in the context of language disorder. As we did not obtain a baseline measure of polysomnography independent of the learning task, we are unable to determine if the SO-slow spindle coupling measures obtained here reflect individual traits or response to learning.

### 4.4 Study limitations and future directions

Several limitations of the current study should be acknowledged. First, the DLD group was substantially smaller than the TD group, limiting our ability to conduct meaningful between-group comparisons. Second, nap sleep may not provide sufficient time for a complete sleep cycle, including deeper stages of non-rapid eye movement (NREM) sleep such as stage N2 and slow-wave sleep (SWS). Because SWS is thought to play a critical role in memory consolidation, and approximately half of the participants did not reach this stage, the effects of sleep observed here may not fully reflect those associated with longer sleep periods. Third, the speech outcome measures focused exclusively on perception of the trained stimuli, providing only a partial view of sleep-related changes in learning. Finally, the study did not include a wake control group, nor did it include baseline polysomnographic measures of sleep. Consequently, although the post-nap improvements observed are consistent with sleep-dependent consolidation, we cannot rule out the possibility that comparable gains would have occurred in the absence of sleep. Furthermore, we are unable to determine if elevated spindle and SO density associated with language difficulties in the present paper reflects individual differences in habitual sleep characteristics, or response to learning.

Future studies should recruit larger and more balanced samples to increase statistical power and support more robust group comparisons. Examining the effects of a full night of sleep would also provide access to a broader range of sleep stages that may contribute to memory consolidation. In addition, future work should incorporate measures of generalization alongside assessments of trained-item perception to provide a more comprehensive evaluation of speech learning outcomes. Finally, the inclusion of a wake control group would allow for a more definitive assessment of the specific contributions of sleep to memory consolidation and performance gains.

## 5 Conclusion

This study examined the relationship between sleep and speech-sound memory consolidation in adults with typical language abilities and in adults with a history of developmental language disorder (DLD). Although identification performance improved following a nap, the absence of a wake control group precludes definitive conclusions regarding the role of sleep in these gains. Instead, sleep should be considered a plausible contributing factor, consistent with prior research demonstrating sleep-related benefits for speech-sound learning.

Interestingly, slow spindle density and slow oscillation were observed with different directional associations with changes to behavioral performance. While these findings may appear inconsistent with the traditional view that sleep spindles and SOs support memory consolidation, a growing body of evidence suggests that negative associations are not uncommon. The present findings contribute to an emerging literature indicating that the relationship between sleep physiology and learning may vary across domains and learner populations.

One possibility is that sleep parameters such as spindle and SO density do not uniformly facilitate memory consolidation across all forms of learning. Furthermore, these neural signatures may serve different functions in individuals with language-learning difficulties than in those with typical language development. Together, these findings challenge simplistic accounts of sleep-dependent memory consolidation and suggest that the relationship between sleep physiology and learning may be more nuanced than previously assumed, particularly in populations with developmental language difficulties.

## Data Availability

The datasets presented in this study can be found in online repositories. The names of the repository/repositories and accession number(s) can be found at: Open Science Framework, https://osf.io/mn7xt/overview?view_only=af1ea22b735846149374cfde0df67f24.

## Footnotes

1
https://sites.google.com/andrew.cmu.edu/tarrlab/stimuli

2
https://osf.io/mn7xt/overview?view_only=af1ea22b735846149374cfde0df67f24
