# Supplementary material for: Speech-sound memory consolidation over a daytime nap in adults with and without developmental language disorder
Source: Front Behav Neurosci. 2026 Sep 30;20:1903903. doi: 10.3389/fnbeh.2026.1903903 (PMC13662794; doi:10.3389/fnbeh.2026.1903903)
Supplement: Supplementary file 1 [file Supplementary_file_1.docx]

Supplementary Material

# 1 Supplementary Data

The main text describes slow and fast spindle densities, and slow oscillation (SO) density, calculated over theoretically-motivated regions of interest (ROI). Here, we explore potential topographical differences between those with developmental language disorder (DLD) and those with typical language (TD). For this set of analyses, we first tested the Student’s *t-*test assumptions of normality and equality of variances via Shapiro-Wilk and Brown-Forsythe tests, respectively, on slow spindle, fast spindle, and DO densities calculated at each channel per participant. In finding that not all channels met assumptions of normality and/or equality of variances, and in considering the uneven sizes of our samples, we proceeded to conduct the Welch’s independent samples *t*-tests at each of the 22 cortical channel locations. These tests compared slow spindle, fast spindle, and SO densities across group (TD vs. DLD). The Holms-Bonferroni correction was applied to control for family-wise error at .05.

For slow spindle density, we did not find any channel to significantly differ across groups (**Table S1**). For fast spindle density with channels Oz, O2, and P8 emerged as significantly different after Holms-Bonferroni correction for family-wise error (**Table S2**, **Figure S1**). These differences were driven by higher fast spindle density in individuals with DLD in these posterior channels. Individuals with DLD also exhibited numerically higher SO density during NREM sleep across most channels; however, these differences were not statistically significant (**Table S3**).

We followed up on the observation of significant differences observed between TD and DLD on fast spindle density in the Oz, O2, and P8 channels, by examining the correlation between fast spindle density and language difficulty in these three channels. To revisit, positive values on the LI index indicate positive identification for DLD. In other words, higher values correspond to greater difficulties with language. Results indicated a significant positive correlation between fast spindle density and the LI index across all three channels after correction (p < 0.05). See **Figure S2** for graphical depiction.

Together, it appears that posterior expression of fast spindles during NREM in posterior regions may be associated with language difficulties. The occipital expression of fast spindles has been linked to poor cognition and impaired memory consolidation, posited to reflect a breakdown in thalamocortical engagement (Zhu et al., 2023). While it is important to observe caution in interpreting the current results in consideration of the small sample of adults with DLD, the current result warrants increased attention to the topography of sleep activity in those with language disorder.

## Table S1

*Comparisons of slow spindle density across TD and DLD*

|  | TD | | DLD | |  |  |  |
| --- | --- | --- | --- | --- | --- | --- | --- |
| Channel | mean | SD | mean | SD | T value | P value | Cohen's d |
| **FP2** | 3.23 | 0.63 | 3.01 | 0.69 | -0.82 | 0.436 | -0.34 |
| **F7** | 3.31 | 0.79 | 2.70 | 1.00 | -1.76 | 0.104 | -0.72 |
| **F3** | 3.51 | 0.84 | 3.12 | 0.48 | -1.25 | 0.24 | -0.51 |
| **F4** | 3.49 | 0.63 | 2.78 | 1.11 | -2.16 | 0.049 | -0.88 |
| **F8** | 3.23 | 0.84 | 3.04 | 0.57 | -0.61 | 0.563 | -0.25 |
| **T7** | 3.05 | 0.76 | 2.93 | 0.44 | -0.41 | 0.697 | -0.17 |
| **C3** | 3.43 | 0.72 | 3.13 | 0.40 | -1.14 | 0.283 | -0.47 |
| **Cz** | 3.09 | 0.63 | 2.94 | 0.35 | -0.65 | 0.54 | -0.26 |
| **C4** | 3.24 | 0.65 | 2.86 | 0.67 | -1.4 | 0.191 | -0.57 |
| **T8** | 3.10 | 0.72 | 3.40 | 0.88 | 0.95 | 0.372 | 0.39 |
| **P7** | 2.90 | 0.70 | 2.52 | 0.65 | -1.36 | 0.205 | -0.55 |
| **P3** | 3.00 | 0.56 | 3.03 | 0.58 | 0.15 | 0.888 | 0.06 |
| **Pz** | 3.17 | 0.56 | 2.95 | 0.69 | -0.89 | 0.4 | -0.36 |
| **P4** | 3.12 | 0.87 | 2.77 | 1.16 | -0.9 | 0.398 | -0.37 |
| **P8** | 3.05 | 0.80 | 3.10 | 0.91 | 0.13 | 0.9 | 0.05 |
| **O1** | 3.08 | 1.19 | 2.61 | 0.67 | -1.08 | 0.309 | -0.44 |
| **Oz** | 3.03 | 1.11 | 2.71 | 0.85 | -0.76 | 0.471 | -0.31 |
| **O2** | 3.00 | 1.03 | 2.58 | 0.74 | -1.09 | 0.304 | -0.45 |
| **CP2** | 3.11 | 0.62 | 2.79 | 0.44 | -1.36 | 0.202 | -0.56 |
| **FC5** | 3.37 | 0.59 | 2.98 | 0.73 | -1.49 | 0.165 | -0.61 |
| **FC1** | 3.54 | 1.09 | 2.97 | 0.47 | -1.45 | 0.175 | -0.59 |
| **FC6** | 3.28 | 0.73 | 2.90 | 0.54 | -1.37 | 0.202 | -0.56 |

**SUPPLEMENTARY TABLE 1.** Parameter estimates and standard deviations (SD) for the group effect on slow spindle density across channels.

**Table S2**

*Comparisons of fast spindle density across TD and DLD*

|  | TD | | DLD | |  |  |  |
| --- | --- | --- | --- | --- | --- | --- | --- |
| Channel | mean | SD | mean | SD | T value | P value | Cohen's d |
| **FP2** | 4.36 | 0.78 | 4.48 | 0.90 | -1.33 | 0.207 | -0.58 |
| **F7** | 4.13 | 0.66 | 4.22 | 1.33 | 0.27 | 0.795 | 0.13 |
| **F3** | 4.43 | 0.82 | 4.79 | 0.73 | -1.25 | 0.229 | -0.52 |
| **F4** | 4.42 | 0.86 | 4.37 | 0.73 | -0.46 | 0.652 | -0.19 |
| **F8** | 4.24 | 0.71 | 4.82 | 0.74 | -2.32 | 0.038 | -0.99 |
| **T7** | 3.72 | 0.90 | 4.20 | 0.77 | -1.19 | 0.252 | -0.49 |
| **C3** | 4.19 | 0.92 | 4.37 | 0.74 | -1.16 | 0.265 | -0.47 |
| **Cz** | 4.03 | 0.94 | 4.19 | 1.08 | -0.70 | 0.496 | -0.31 |
| **C4** | 4.15 | 0.99 | 4.26 | 0.87 | -1.08 | 0.298 | -0.45 |
| **T8** | 3.72 | 0.90 | 4.39 | 0.48 | -1.97 | 0.061 | -0.74 |
| **P7** | 3.52 | 0.88 | 3.85 | 0.84 | -1.79 | 0.095 | -0.75 |
| **P3** | 3.80 | 0.96 | 4.24 | 0.88 | -1.48 | 0.161 | -0.62 |
| **Pz** | 3.98 | 0.98 | 4.16 | 0.81 | -1.51 | 0.151 | -0.62 |
| **P4** | 3.66 | 0.99 | 3.94 | 0.88 | -1.00 | 0.335 | -0.41 |
| **P8** | 3.40 | 0.80 | 4.21 | 0.72 | -3.16 | 0.006* | -1.32 |
| **O1** | 3.30 | 1.08 | 4.13 | 0.98 | -2.69 | 0.017 | -1.12 |
| **Oz** | 3.21 | 1.04 | 4.17 | 0.46 | -3.86 | < .001* | -1.42 |
| **O2** | 3.23 | 1.06 | 4.05 | 0.50 | -3.29 | 0.003* | -1.22 |
| **CP2** | 4.12 | 1.10 | 4.16 | 0.96 | -1.12 | 0.279 | -0.46 |
| **FC5** | 4.26 | 0.88 | 4.36 | 0.74 | 0.14 | 0.892 | 0.06 |
| **FC1** | 4.55 | 1.06 | 4.28 | 0.71 | 0.78 | 0.442 | 0.31 |
| **FC6** | 4.16 | 0.79 | 4.35 | 0.81 | -0.71 | 0.488 | -0.30 |

**SUPPLEMENTARY TABLE 2.** Parameter estimates and standard deviations (SD) for the group effect on fast spindle density across channels. *denotes statistical significance at .05 level after Holms-Bonferroni correction for family-wise error rate.

**Table S3**

*Comparisons of slow oscillation (SO) density across TD and DLD*

|  | TD | | DLD | |  |  |  |
| --- | --- | --- | --- | --- | --- | --- | --- |
| Channel | mean | SD | mean | SD | T value | P value | Cohen's d |
| **FP2** | 5.14 | 1.09 | 5.18 | 1.00 | 0.09 | 0.933 | 0.04 |
| **F7** | 5.07 | 1.04 | 5.01 | 1.13 | -0.13 | 0.901 | -0.06 |
| **F3** | 5.06 | 0.88 | 5.20 | 1.04 | 0.34 | 0.743 | 0.15 |
| **F4** | 5.12 | 1.02 | 4.98 | 0.77 | -0.39 | 0.701 | -0.16 |
| **F8** | 4.96 | 1.10 | 5.10 | 1.11 | 0.31 | 0.759 | 0.13 |
| **T7** | 5.14 | 1.06 | 5.27 | 1.04 | 0.29 | 0.775 | 0.12 |
| **C3** | 5.00 | 0.96 | 5.17 | 1.06 | 0.40 | 0.699 | 0.17 |
| **Cz** | 5.13 | 0.88 | 5.27 | 1.00 | 0.33 | 0.744 | 0.15 |
| **C4** | 5.04 | 1.04 | 5.07 | 1.09 | 0.07 | 0.945 | 0.03 |
| **T8** | 5.07 | 1.05 | 5.10 | 1.00 | 0.08 | 0.941 | 0.03 |
| **P7** | 5.10 | 1.04 | 5.32 | 1.02 | 0.51 | 0.621 | 0.21 |
| **P3** | 5.12 | 1.06 | 5.24 | 1.23 | 0.24 | 0.814 | 0.10 |
| **Pz** | 5.16 | 0.81 | 5.51 | 0.90 | 0.93 | 0.371 | 0.40 |
| **P4** | 5.29 | 1.07 | 5.53 | 1.01 | 0.56 | 0.586 | 0.23 |
| **P8** | 5.26 | 1.08 | 5.06 | 1.01 | -0.44 | 0.667 | -0.18 |
| **O1** | 5.35 | 1.17 | 5.24 | 0.94 | -0.26 | 0.796 | -0.11 |
| **Oz** | 5.43 | 1.09 | 5.13 | 0.90 | -0.72 | 0.481 | -0.30 |
| **O2** | 5.42 | 1.13 | 5.26 | 0.95 | -0.37 | 0.715 | -0.15 |
| **CP2** | 5.16 | 0.87 | 5.33 | 1.13 | 0.37 | 0.716 | 0.17 |
| **FC5** | 5.05 | 0.91 | 5.26 | 0.99 | 0.52 | 0.612 | 0.23 |
| **FC1** | 5.14 | 0.92 | 5.25 | 1.03 | 0.24 | 0.815 | 0.10 |
| **FC6** | 5.03 | 1.15 | 5.07 | 1.01 | 0.10 | 0.921 | 0.04 |

**SUPPLEMENTARY TABLE 3.** Means and standard deviations (SD) for the group effect on slow oscillation density (SO) across channels. SO density was not found to significantly differ across groups.

**Figure S1**

*Topographical comparison between TD and DLD on spindle and slow oscillation (SO) density*

A. Slow spindle density	B. Fast spindle density	C. SO Density

**SUPPLEMENTARY FIGURE 1.** Topographical maps of (A) slow spindle (B) fast spindle, and (C) slow oscillation (SO) average density differences between DLD and TD groups. Fast spindle density was significantly higher in DLD at P8, Oz, and O2, p < 0.05 (channels with uncorrected p-values below .05 are marked in white).

**Figure S2**

*Correlation between fast spindle density in posterior channels and language difficulty*

**SUPPLEMENTARY FIGURE 2.** A representative correlation between posterior (P8) fast spindle density and the language impairment (LI) index, wherein greater values indicate greater difficulties with language. Fast spindle activity expressed at P8, O2, and Oz are positively associated with greater language difficulty.
